# Evaluation of ergosterol content in the air of various environments

**DOI:** 10.1007/s10453-014-9344-4

**Published:** 2014-06-06

**Authors:** Beata Gutarowska, Justyna Skóra, Katarzyna Pielech-Przybylska

**Affiliations:** Institute of Fermentation Technology and Microbiology, Lodz University of Technology, Wólczańska St. 171/173, 90-924 Lodz, Poland

**Keywords:** Ergosterol, Bioaerosol, Microorganisms, Indoor air, Outdoor air

## Abstract

The aim of the study was to compare the content of ergosterol in different microorganisms (bacteria, yeasts and moulds) isolated from the air as well as in six species of moulds in their different morphological forms—live mycelium, dead mycelium, and spores. Evaluation of the level of mould contamination of the air in various places using culture method and ergosterol determination was also performed. The analysis of ergosterol was carried out by gas chromatography equipped with flame ionisation detector. For evaluation of the results, analysis of variance and multiple comparison test were used. The quantity of ergosterol in the spores of various species of mould was in the range 1.9–9.4 pg/spore. The presence of yeasts and bacteria in the air does not significantly affect ergosterol concentration, in view of the low content of that sterol in their cells (max. 0.009 pg/cell for bacteria and 0.39 pg/cell for yeast). An ergosterol concentration above 1 ng per m^3^ can be considered an indicator of excessive mould contamination of indoor air. Based on determination of ergosterol in the air of mouldy rooms the result obtained may be compared with the culture method, due to the 1,000 times higher concentration of ergosterol in the mycelium compared with spores. However, in the analysis of outdoor air, in view of the presence of mould mainly in the form of spores and the degradation of ergosterol by UV radiation, analysis of that compound may indicate a lower level of contamination compared with the culture method.

## Introduction

Ergosterol is a basic sterol forming the cellular membranes of moulds and yeasts and accounts for (depending on growth phase) 10–80 % of all sterols present in the mycelium. Hence, it is easy to detect among other cell components (Seitz et al. 1979).

The first studies on the use of ergosterol as an indicator of the presence of mould in the environment were done by Seitz et al. (1977, 1979). Ergosterol has most often been associated with the occurrence of moulds and yeasts in various natural environments (Newell 1991). It does not occur in the air, attention has been drawn to the low content of ergosterol in the cells of algae and protozoa, bacteria and pollen (Raederstorff and Rohmer 1987; Peeler et al. 1989); hence, the air background is free of ergosterol, in animal and fungal cells (e.g. in mites, moulds present in dust) it occurs as provitamin D (Holick 1984).

The average ergosterol content in one mould spore has been estimated at 0.11–5.1 pg for the species *Aspergillus versicolor*, *Penicillium brevicompactum*, *Cladosporium cladosporioides*, *Aureobasidium pullulans* and *Stachybotrys chartarum* (Miller and Young 1997; Pasanen et al. 1999). However, different researchers have found different ergosterol concentration values for the same mould species; the reason for this may be the different measuring procedures used (some entailed analysis of the spores alone, while others included the ergosterol in the mycelium as well as in spores). Ergosterol occurs in all moulds morphological forms—spores, living and dead mycelium—and in every phase of growth, including in submicron and unculturable forms. Since ergosterol is detected in non-living forms, it may serve as an indicator of earlier moulds infestation.

There are some methods for determining the level of moulds infestation of air and construction materials in buildings, of which the most commonly used are culture methods and the determination of ergosterol, β-d glucan, volatile compounds (MVOCs) and mycotoxins (Larssen and Frisvad 1994; Nielsen et al. 1999; Nielsen and Madsen 2000; Chew 2001). Among these method, measurement of ergosterol is faster and easier; it takes several hours, compared with the days or weeks required for culture analysis and the complicated procedures for determination of MVOCs, mycotoxins, toxicity, molecular analysis, and other specialist measurements. It has an advantage over the culture method in that it also detects unculturable forms not capable of growing on specified microbiological media.

There are some methods of ergosterol determination—HPLC, GC (with FID, MS or MS–MS), spectrophotometric UV or NIR; these can be used depending on the laboratory’s equipment, although the sensitivity depends on the method of determination (the most sensitive are GC–MS–MS and HPLC: 1 ng/µl) (Jirout et al. 2010).

Comparative analyses of various methods for evaluating the presence of moulds in building environments and plant products have shown the greatest correlation to be between ergosterol determination and a quantitative culture method (*R* ^2^ = 0.84–0.94), while the lowest correlations were obtained in comparing the method of MVOC determination (ng/h) and the culture method (*R* ^2^ = 0.35), or CO_2_ content (m/h) and the culture method (*R* ^2^ = 0.57) (Börjesson et al. 1990; Gutarowska and Żakowska 2002). The correlation between the content of cellular components (ergosterol, β-d glucan) or mould numbers (cfu) and the level of metabolites produced by moulds (MVOC, ATP, CO_2_, mycotoxins) may be low in many cases because metabolite synthesis is a strain feature, dependent to a large extent on environmental conditions, the phase of growth and the presence of coexisting microorganisms. For the above reasons, the method of ergosterol determination also takes on great practical significance.

The ergosterol method is widely recommended for evaluation of moulds and yeasts in the soil and for the monitoring of soil bioremediation, since the stability of that compound has been found not to be significantly affected by heavy metal impurities (Barajas-Aceves et al. 2002). Ergosterol has been used as a marker of the quantity of moulds and yeasts in the soil during particular phrases of composting (Montgomery et al. 2000; Suberkropp 2001; Stahl and Parkin 1996). It has also been used for measuring the risk of contamination of food with toxin-forming moulds (Saxena et al. 2001; Gutarowska and Żakowska 2010). DeLuca et al. (1995) showed that the oxidation of ergosterol may be a signal for the synthesis of aflatoxins. Moreover, ergosterol has been used to measure mould infestation of construction materials (Pasanen et al. 1999; Nielsen and Madsen 2000; Gutarowska and Żakowska 2002; Hippelein and Rügamer 2004) and to estimate numbers of moulds in household dust (Miller et al. 1988; Axelsson et al. 1995). Ergosterol was detected in household dust in concentrations of 0.7–45 μg/g.

In spite of these numerous applications, attention has been drawn to the instability of this compound in the natural environment (Newell et al. 1989; Stahl and Parkin 1996; Montgomery et al. 2000). Mille-Lindblom et al. (2004) showed that it underwent significant degradation (43 %) under the action of light. Also ergosterol synthesis has been found to be significantly influenced by phase of moulds growth, ageing, and environmental parameters (media, pH, temperature) (Torres et al. 1992; Gutarowska and Żakowska 2009). Studies have shown that on the death of mould the ergosterol content may fall by as much as 34 % (Mille-Lindblom et al. 2004).

Ergosterol has been recommended previously as a marker for determination of the presence of moulds in indoor and outdoor air (Miller et al. 1988; Miller and Young 1997; Pasanen et al. 1999; Robine et al. 2005). The limit for determination of ergosterol by the HPLC method, using a rotating cup system for air sampling, has been put at 0.4 ng/m^3^, equivalent to 150 spores/m^3^ of air (Robine et al. 2005). However, the concentrations of ergosterol in the air measured by various authors range from 0.01 to 194 ng/m^3^ (Miller et al. 1988; Miller and Young 1997; Pasanen et al. 1999; Robine et al. 2005). Determination of ergosterol in the air has been performed chiefly for mould spores, using appropriate methods of measurement. However, bioaerosols contain different morphological forms of microorganisms apart from spores, including fragments of living and dead mycelium.

By testing the content of ergosterol in the air, it is possible to estimate the total biomass of moulds and yeasts which may affect the human respiratory system, including submicron forms, unculturable forms, spores, and inactive mycelium, which may be carriers of mycotoxins and allergens.

The goal of the present study was to compare the content of ergosterol in the cells of bacteria, yeasts and moulds isolated from the air and in moulds in different morphological forms—live mycelium, dead mycelium and spores. Tests were also performed to evaluate the level of mould contamination of the air in various places using the ergosterol method and a culture method. This analysis provides a basis on which to address the question of whether ergosterol can be a useful marker of the presence of mould in the air in different morphological forms and whether ergosterol levels differ depending on the degree of contamination with cultivated moulds.

## Materials and methods

### Tested environments

Tests of mould contamination of the air were carried out in eight types of environment: homes with visual moulds growth on walls, control homes without signs of mould infestation, school premises, hospital premises, archive and library storerooms, museum storerooms, production halls in a pharmaceutical plant, and outdoor air. The number of tested sites and a description of the rooms are given in Table 1.

**Table 1:** Description of places tested

Places tested	Number of tested objects	Description
Mould-infested residential rooms	10	Signs of mould on walls. Tests performed in bathrooms (5), kitchens (3) and living rooms (2). Rooms without air conditioning system. Tested in months: October–May Air humidity in homes RH = 50–75 %, temp. 15–20 °C
Residential rooms without signs of mould	10	Tests performed in bathrooms (5), kitchens (4) and living rooms (1). Rooms without air conditioning system. Tested in months: October – May Air humidity in homes RH = 30–60 %, temp. 18–25 °C
Rooms in schools	3	A primary school classroom (1; for 20 pupils), and college lecture halls (2; numbers of students 25 and 50). Rooms without air conditioning system. Tested in months: October–May Air humidity RH = 35–50 %, temp. 20–22 °C
Rooms in hospitals	3	An operating room (1) and wards (2; number of beds 6). Rooms without air conditioning system. Tested in months: October–May Air humidity RH = 30–40 %, temp. 19–22 °C
Production halls in a pharmaceutical plant	2	Rooms in controlled “grey” zone, hall with equipment for grinding of bacteriostatic substance (1), herb packing hall (1). Rooms with air conditioning system. Tested in months: October–May Air humidity RH = 42–50 %, temp. 18–20 °C
Rooms in libraries and archives	4	Library storerooms (2), archive storerooms (2). Rooms without air conditioning system. Tested in months: October–May Air humidity RH = 27–51 %, temp. 15–23 °C
Rooms in museums	4	Storerooms containing martyrological objects (1), works of folk art (1), textile objects (1), a conservation laboratory (1). Rooms without air conditioning system. Tested in months: October–May Air humidity RH = 30–50 %, temp. 19–22 °C. Numerous visible symptoms of biodeterioration of museum objects
Outdoor air	6	Tests of outdoor air were performed every 2 months in the centre of a city (population c.1 million). Tested in months: October–May

The temperature and humidity of the air in the tested environments were determined using a PWT-401 hygrometer (Elmetron, Poland).

The tests were performed in different places in each room, in six repetitions, at times of greatest activity of residents or staff, to obtain average results approximately reflecting an authentic state under the specified conditions.

### Microorganisms

The microorganisms used in the study were isolated from the indoor air of residential and public buildings and of a pharmaceutical plant, and from outdoor air (Table 1). The tests involved microorganisms for which the frequency of isolation in the air of the tested environments was >30 %. Following identification (using the methodology described below), the strains were used to determine the content of ergosterol in the cells (bacteria, yeasts and moulds) and in different morphological forms (moulds). These strains were contained in the LOCK Collection of Pure Cultures (ITFM UT). The types of microorganisms used in the tests and the places from where they were isolated are given in Table 2.

**Table 2:** Microorganisms used in the study

Microorganisms	Place of isolation
Bacteria	
*Bacillus subtilis*	Hospital room
*Micrococcus flavus*	School room
Yeast	
*Rhodotorula glutinis*	Pharmaceutical plant
*Candida glabrata*	Home without signs of mould
Moulds	
*Alternaria alternata*	Outdoor air
*Aspergillus niger*	Mould-infested home
*Aspergillus versicolor*	Mould-infested home
*Cladosporium cladosporioides*	Mould-infested home
*Penicillium chrysogenum*	Pharmaceutical plant
*Penicillium expansum*	School room

### Mycological analysis of the air

Mycological contamination of the air was determined using an MAS-100 Eco Air Sampler (Merck, Germany) with 100 l/min flow rate. Samples of 50 and 100 l of air were taken during 30s and 60s on an MEA medium (Malt Extract Agar, Merck, Germany) with chloramphenicol (0.1 %) for determination of the total number of fungi and for their isolation. Air samples were taken at three points in each place, in two repetitions. Next, the samples were incubated at 27 ± 2 °C for 5 days (according to EN 13098:^2000^). After incubation, the growing colonies were counted, and the result, taking into account the volume of sampled air, was converted to units of cfu/m^3^ (for bacteria and yeasts means cells, for moulds—spores and fragments of mycelium present in the air). The final result was taken to be the arithmetic mean of all repetitions for a given place.

### Isolation and identification of bacteria and yeasts

Air samples for isolation of bacteria were taken, using the method described above, on a TSA medium (Tryptic Soy Agar, Merck, Germany) with nystatin (0.2 %) and incubated at 30 ± 2 °C for 48 h, yeasts were isolated on MEA medium at 27 ± 2 °C for 72 h (according to EN 13098:^2000^). The pure cultures of bacteria and yeast (four strains were chosen for the next step, with frequency of occurrence >30 %) were characterised macro- and microscopically, and then, selected diagnostic features were tested for bacteria: Gram-colouring, catalase test, and oxidase test (Microbiologie Bactident Oxydase, Merck, Germany). For bacteria, API tests were performed (BioMérieux, France): API 50 CH, API STAPH. For yeasts, diagnosis was performed using API C AUX. Identification agreement for the tested strains was higher than 99 %.

### Identification of moulds

Identification of all moulds was performed based on macroscopic and microscopic observations of strains cultured on CYA medium (Difco, USA) and YES (Yeast Extract with Supplements), with the use of taxonomic keys (Samson et al. 2000; Flannigan et al. 2001). For subsequent tests, six strains were used for which the frequency of isolation was >30 %.

### Ergosterol determination in the air

Air samples for ergosterol determination were taken by a filtration method using an AirPort MD8 sampler (Sartorius, Germany) with 30 l/min flow rate. Samples of volume 1000 l (two repetitions, three points in each place) during 2000s were taken on sterile gelatine filters (pore diameter 0.3 µm, Sartorius, Germany). The analytical procedure for quantitative determination of ergosterol was based on the modified method given by Seitz et al. (1977). Ergosterol was extracted using methanol and was shaken for 30 min at 150 × *g*. Samples were saponified with KOH, after which extraction was performed using hexane and a hexane layer was separated off. The sample was evaporated on a vacuum evaporator and suspended in methanol. Chromatographic analysis (GC-FID) was carried out using GC apparatus (Agilent 6890 N HP, USA). Compounds were separated using an HP-5 capillary column (Agilent, USA) of dimensions 30 m × 0.32 mm × 0.25 μm with a non-polar stationary phase in the form of 5 % phenyl–95 % methyl polysiloxane. The carrier gas used was helium, with a flow rate through the column of 2 ml/min. The temperatures of the injector (S/SL—split/splitless injector) and detector (FID—flame ionisation detector) were 250 and 280 °C, respectively. Samples were injected without split (purge activation time: 1 min). The analysis was performed in programmed temperature conditions: initial temperature 150 °C (1 min), then increased at 15 °C/min to 310 °C (5 min). Quantitative analysis was performed using an external standard (external calibration) method, using Agilent ChemStation (USA) software. A calibration curve was built for ergosterol concentrations ranging between 0.35 and 3.5 µg/ml. The curve was linear in the concentration range considered *R* ^2^ being 0.992.

Sensitivity of GC with flame ionisation detector was evaluated by calculating the values of limit of detection (LOD) and limit of quantitation (LOQ). LOD and LOQ were calculated by method based on the standard deviation of the response and the slope of the calibration curve at levels approximating the LOD (ICH 1996).

### Ergosterol determination in microorganism cells

The determination of ergosterol content in the cells of microorganisms was performed on prepared samples of biomass containing a known number of cells. For this purpose, biological material was taken from culture on a medium in conditions suitable for the microorganisms in question (bacteria—TSA, temp. 30 ± 2 °C for 48 h; yeasts—MEA, temp. 27 ± 2 °C for 72 h). The material was taken from three Petri dishes with media into 100 ml methanol, and the biomass was then subjected to extraction and determination of ergosterol (using the extraction and measurement methodology described above).

In the case of moulds, the ergosterol content was determined in the spores, which were collected from the surface of three Petri dishes with MEA medium following culture for 7–10 days at 27 ± 2 °C (so that abundant spore formation took place). The material was collected carefully into 100 ml methanol and filtered through three layers of gauze to remove mycelium fragments. Filtration was done using vacuum pump and Buchner funnel. After each filtration, the presence of the spore fraction was checked microscopically using light microscope. The filtration was repeated to separate only spore fraction without mycelium fragments (three times). The purity and density of cells was determined microscopically in a Thoma chamber based on 20 measurements (20 samples with 10 areas of the chamber were counted).

The experiment was performed three times. Cells densities were in the range 1.7–3.5 × 10^12^ cfu/ml for bacteria, 3.5–4.8 × 10^10^ cfu/ml for yeasts, and 1.5–6.8 × 10^8^ cfu/ml for moulds. The ergosterol content, taking into account the number of cells in the extract, was recorded in units of pg/cell (bacteria, yeast) or pg/spore (moulds).

Ergosterol content was also determined in mould in different morphological forms: living mycelium, inactive mycelium, and spores (converted to dry mass). Living mycelium of particular species was obtained following 40 h of incubation in liquid Malt Extract Bouillon medium (MEB, Merck, Germany) at 27 ± 2 °C and with shaking at 150 × *g.* After visible hyphae were obtained, the mycelium was separated from the medium by filtration, and then, a sample of biomass of known weight was subjected to extraction using methanol and to determination of ergosterol (in the conditions described above). Additionally, a fragment of mycelium was dried to constant mass (using an MAC 110/NH moisture balance, Radwag, Poland). Taking account of the dry mass content of the mycelium, the results were stated in units of mg ergosterol/g dry mass. Inactive mycelium was prepared similarly, except that after separation its growth was suppressed by pasteurisation (at 100 °C for 30 min.) and it was then stored in darkness for 2 weeks. The mycelium fraction was checked also microscopically (20 preparations in 10 samples), it was observed only mycelium fragments in the form of pellets. After this time, ergosterol extraction was performed and the dry mass of mycelium was determined.

Material for analysis of ergosterol in the dry mass of spores was taken from ten Petri dishes with MEA medium into 100 ml distilled water, by the same technique as described above. Then, the water was evaporated until the spore dry mass was obtained. This was subjected to extraction with methanol, followed by determination of ergosterol. Results were recorded in units of ng/mg spore dry mass.

All measurements were repeated in three independent experiments.

### Statistic analysis

Statistical calculations were performed using STATISTICA 6.0 software (Statsoft, USA). The obtained results were evaluated using one-way and two-factor analysis of variance (ANOVA) at the significance level 0.05. When statistical difference was detected (*p* < 0.05), means were compared by the Tukey’s test at significance level 0.05.

## Results

Analysis concerned the content of ergosterol in three groups of microorganisms: bacteria, yeasts, and moulds (Table 3). No statistical differences were found for ergosterol concentration in the tested strains of bacteria (*p* > 0.05), which amounted to 0.008–0.009 pg/cell. However, significantly different (*p* < 0.001) ergosterol concentrations were found in yeasts, in which the content of that compound lay in the range 0.125–0.397 pg/cell.

**Table 3:** Ergosterol content in the cells of various microorganisms

Microorganisms	Ergosterol content (pg/spore^a^ or pg/cell^b^) (*N* = 3)
Bacteria	
*Bacillus subtilis*	*X*: 0.008 SD: 0.006 a
*Micrococcus flavus*	*X*: 0.009 SD: 0.015 a
Yeast	
*Rhodotorula glutinis*	*X*: 0.125 SD: 0.005 a
*Candida glabrata*	*X*: 0.397 SD: 0.022 b
Moulds	
*Alternaria alternata*	*X*: 3.800 SD: 0.073 b
*Aspergillus niger*	*X*: 2.125 SD: 0.012 a
*Aspergillus versicolor*	*X*: 1.923 SD: 0.050 a
*Cladosporium cladosporioides*	*X*: 9.390 SD: 0.152 e
*Penicillium chrysogenum*	*X*: 5.033 SD: 0.019 c
*Penicillium expansum*	*X*: 7.627 SD: 0.087 d

*X* mean, *SD* standard deviation, *N* number studied samples means (among groups of microorganisms) with the same letter are not significantly different (one-way ANOVA, *p* < 0.05; Tukey’s test, *p* < 0.05)

^a^Mould

^b^Bacteria, yeast

Based on single-factor analysis of variance, statistically significant differences were also found in the ergosterol concentration determined in mould spores (*p* < 0.001). In statistical analysis using the Tukey’s test, at a significance level of 0.05, no statistical differences were found (*p* > 0.05) between the concentration of ergosterol in spores of the strain *Aspergillus niger* (2.125 pg/spore) and spores of the strain *Aspergillus versicolor* (1.923 pg/spore). The results for ergosterol concentration in the spores of the remaining four strains of mould are statistically different (*p* < 0.05).

Compared ergosterol concentration between bacteria, yeast, and moulds, the lowest level is found in the cells of bacteria (*p* < 0.001), while a 100 times higher ergosterol concentration was found in the cells of yeast and a 1,000 times higher concentration in mould spores.

 Ergosterol content is not correlated with the sizes of mould spores—the largest spores, those of *Alternaria alternata* (18–83 × 7–18 μm according to Samson et al. 2000), have an ergosterol content of 3.8 pg/spore, while the smallest, those of *Penicillium expansum* (3.0–3.5 × 2.5–3.0 μm according to Samson et al. 2000), contain 7.5 pg/spore.

Further tests were performed to determine the concentration of ergosterol in various morphological forms (living mycelium, dead mycelium and spores) of six mould strains. Analysis of variance showed significant differences (Table 4)—between morphological groups of the tested strains, between mould strains, and in tests of the interaction of both factors. To test this assumption more accurately, a Tukey’s multiple comparison test was carried out, at a significance level of 0.05 (Table 5).

**Table 4:** Ergosterol content in morphological forms of moulds isolated from indoor air

Mould species	Ergosterol content (*N* = 3)		
	Living mycelium (mg g^−1^ dry weight of mycelium)	Non-living mycelium (mg g^−1^ dry weight of mycelium)	Spores (ng mg^−1^ dry weight of spores)
*Alternaria alternata*	*X*: 4.24 SD: 0.11 bA	*X*: 3.16 SD: 0.07 bA	*X*: 1.57 SD: 0.02 aAB
*Aspergillus niger*	*X*: 3.95 SD: 0.05 bA	*X*: 4.02 SD: 0.08 bA	*X*: 1.53 SD: 0.47 aAB
*Aspergillus versicolor*	*X*: 3.86 SD: 0.25 bA	*X*: 5.40 SD: 0.13 bB	*X*: 1.76 SD: 1.03 aAB
*Cladosporium cladosporioides*	*X*: 9.92 SD: 0.10 cB	*X*: 7.89 SD: 0.17 bC	*X*: 2.42 SD: 0.09 aB
*Penicillium chrysogenum*	*X*: 4.76 SD: 0.31 bA	*X*: 4.06 SD: 0.25 bA	*X*: 2.23 SD: 0.35 aB
*Penicillium expansum*	*X*: 5.93 SD: 0.17 cC	*X*: 3.93 SD: 0.09 bA	*X*: 1.07 SD: 0.72 aA

*X* mean, *SD* standard deviation, *N* number studied samples

a–c means with the same small letter in the same row are not significantly different (Tukey’s test; *p* < 0.05)

A–C means with the same capital letter in the same column are not significantly different (Tukey’s test; *p* < 0.05)

**Table 5:** Effects of the mould species and morphological forms of moulds on the content of ergosterol

Factor	Sum of squares SS	Degrees of freedom df	Mean square MS	*F*	*p*
Mould species	86.2887	5	17.2577	135.023	<0.001
Morphological forms of moulds	137.3685	2	68.6843	537.381	<0.001
Mould species × Morphological forms of moulds	41.6169	10	4.1617	32.561	<0.001
Error	4.6013	36	0.1278		

Two-factor analysis of variance (ANOVA, *p* < 0.05)

The Tukey’s procedure showed that there are statistically significant differences in ergosterol concentration depending on the morphological form of mould, when the level in spores is compared with the level in living mycelium and dead mycelium, irrespective of which mould strain is tested. The spores of all tested strains of mould contained the lowest concentration of that compound (from 1.07 to 2.42 ng/mg dry weight of spores).

However, comparing the ergosterol concentrations in living and dead mycelium, the differences in ergosterol content are found to be statistically significant (*p* < 0.05) only when results are compared for the strain *Cladosporium cladosporioides* (9.92 mg/mg dry weight of mycelium) and for *Penicillium expansum* (5.93 mg/mg dry weight of mycelium).

The ergosterol content in living and dead mycelium of mould species: *Alternaria alternata*, *Aspergillus niger*, *Aspergillus versicolor*, and *Penicillium chrysogenum* does not differ markedly (*p* > 0.05) and ranges from 3.93 to 5.40 mg/g dry mass of mycelium (Table 4).

Tests of moulds contamination of the air in different environments showed that in places with visual moulds growth on walls and with raised mould numbers in the air as determined by the culture method (cfu/m^3^), a higher level of ergosterol was also found (mould-infested rooms, museum storerooms containing objects with biodeterioration). In mould-infested rooms, the number of moulds reached 3.1–3.6 × 10^3^ cfu/m^3^, and ergosterol content ranged from 1.4 ng/m^3^ (museum rooms) to 2.6 ng/m^3^ (home with visual moulds growth on walls) (Table 6). There was a wide variety of mould species in these premises. Dominant were moulds of the genera *Aspergillus* and *Penicillium*, the most common species being *Asperillus versicolor*, *A. niger*, *A. flavus*, *Penicillium brevicompactum*, *P. digitatum*, *P. carneum*, *P. paneum* and *P. polonicum*. Other species isolated from the air in museum storerooms and homes with visual moulds growth on walls were *Alternaria alternata*, *Cladosporium cladosporoides*, *C. macrocarpum*, *Rhizopus nigricans* and *Trichoderma viride.*

**Table 6:** Mycological contamination of the air in various environments

Places/number of places tested	Ergosterol content (ng m^−3^ air)	Number of fungi (cfu m^−3^ air)	Dominant fungus species^a^
Mould-infested rooms *N* = 10	*X*: 2.60 SD: 1.30 Max: 6.42 Min: 1.40	*X*: 3.1 × 10^3^ SD: 5.2 × 10^3^ Max: 2.5 × 10^4^ Min: 1.4 × 10^2^	*Asperillus versicolor, A. niger, A. flavus, Trichoderma viride, Penicillium brevicompactum, P. digitatum, Alternaria alternata, Cladosporium cladosporioides*
Residential rooms without signs of mould *N* = 10	*X*: 0.53 SD: 0.26 Max: 1.30 Min: 0.30	*X*: 2.1 × 10^2^ SD: 1.7 × 10^2^ Max: 7.2 × 10^2^ Min: 4.0 × 10^1^	*Alternaria alternata, Cladosporium cladosporioides, C. herbarum, Penicillium brevicompactum, P. expansum*
School rooms *N* = 3	*X*: 0.72 SD: 0.15 Max: 1.29 Min:0.45	*X*: 5.9 × 10^2^ SD: 3.0 × 10^2^ Max: 1.2 × 10^3^ Min: 1.5 × 10^2^	*Penicillium chrysogenum, Rhizopus nigricans, Alternaria alternata, Cladosporium herbarum, Penicillium brevicompactum, P. expansum*
Hospital rooms *N* = 3	*X*: 0.86 SD: 0.12 Max: 1.18 Min: 0.54	*X*: 1.8 × 10^2^ SD: 8.9 × 10^1^ Max: 3.1 × 10^2^ Min: 7.8 × 10^1^	*Penicillium chrysogenum, Alternaria alternata, A. niger, Cladosporium cladosporioides*
Production rooms in a pharmaceutical plant *N* = 2	*X*: 0.67 SD: 0.18 Max: 0.70 Min: 0.51	*X*: 2.3 × 10^2^ SD: 1.0 × 10^2^ Max: 3.6 × 10^2^ Min: 8.7 × 10^1^	*Cladosporium herbarum, Alternaria alternata, Sporotrichum* sp., *Penicillium chrysogenum*
Rooms in libraries and archives *N* = 4	*X*: 0.62 SD: 0.14 Max: 0.72 Min: 0.58	*X*:1.6 × 10^2^ Min:5.0 × 10^1^ Max:3.6 × 10^2^ SD:1.5 × 10^2^	*Cladosporium macrocarpum, C. herbarum, Penicillium crustosum*, *P. carneum, Aspergillus niger*
Rooms in museums *N* = 4	*X*: 1.38 SD: 0.35 Max: 1.83 Min: 0.53	*X*: 3.6 × 10^3^ Min: 7.5 × 10^1^ Max: 1.4 × 10^4^ SD: 6.9 × 10^4^	*Rhizopus nigricans, Aspergillus versicolor, A. niger, Cladosporium macrocarpum, Penicillium carneum, P. paneum*, *P. polonicum*
Outdoor air *N* = 6	*X*: 1.05 SD: 0.16 Max: 4.20 Min: 0.25	*X*: 5.4 × 10^3^ SD: 4.9 × 10^2^ Max: 7.9 × 10^3^ Min: 8.0 × 10^1^	*Acremonium strictum, Cladosporium herbarum, C. cladosporioides, Alternaria alternata*

*X* mean, *SD* standard deviation

^a^Where the frequency of isolation was higher than 30 % of all microorganisms

The highest number of moulds was recorded in outdoor air (5.4 × 10^3^ cfu/m^3^), where the ergosterol content reached 1.1 ng/m^3^. Certain species of mould were characteristic of that environment, the dominant ones being *Alternaria alternata*, *Cladosporium* sp. and *Acremonium* sp.

In rooms where the number of moulds in the air was found to be at a low level, ranging from 1.6 × 10^2^ to 5.9 × 10^2^ cfu/m^3^ (rooms in libraries, archives, hospitals, schools, homes without mould problems, and production halls in a pharmaceutical plant), the ergosterol content did not exceed 1 ng/m^3^ (it lay in the range 0.53–0.86 ng/m^3^). The dominant moulds in these premises belonged to the same species: *Cladosporium macrocarpum*, *C. herbarum*, *C.cladosporioides*, *Penicillium brevicompactum*, *P. chrysogenum*, *P. crustosum*, *P. carneum, Aspergillus niger*, *Alternaria alternata.*

## Discussion

Variation was found in the content of ergosterol in the cells of different microorganisms: bacteria had the lowest content, and moulds the highest—observed differences between these groups were significantly (*p* < 0.001). The levels of ergosterol in the cells of bacteria and yeasts and in mould spores indicate that ergosterol can be used for airborne mould determination only. The concentration of ergosterol in yeast cells is ten times smaller than for moulds, and their numbers in the air do not normally exceed 10^1^ cfu/m^3^ (for example 5.2 × 10^1^ cfu/m^3^ according to Robertson 1998), and hence, they do not make a significant contribution to the total pool of ergosterol measurable in the air.

Analysing the ergosterol content in spores as obtained in the present work and in earlier studies (Miller and Young 1997; Pasanen et al. 1999), the greatest difference was found in the case of the mould genera *Aureobasidium* and *Acremonium,* high differences in ergosterol content were also obtained among species of the genus *Penicillium*. Among species of the genus *Aspergillus*, the difference in ergosterol content was much smaller. Differences were also found in ergosterol content in strains of the same species as given by different authors. Differences between strains of *A. versicolor* and *A. niger* were small, but among strains of *C. cladosporioides* and *P. brevicompactum* that the differences were at the high level. These differences may result from the different authors’ use of different analytical methods and testing of differing morphological fractions (e.g. mixtures of spores and mycelium), but they are primarily linked to genus, species and strain features.

Considering an average level of mould presence in the air of 10^2^ cfu/m^3^ and assuming the value of ergosterol concentration in spores to be in the range 2–10 pg, with the additional assumption that moulds occur solely in the form of spores we obtain a value of 200–1000 pg/m^3^ air (0.2–1 ng/m^3^). Earlier research by Robine et al. (2005) found the level of detection of ergosterol in the air to be 0.4 ng/m^3^; at that level we are able to detect ergosterol only in the case of contamination by certain mould species, e.g. *Cladosporium* *cladosporioides* (ergosterol content 9.4 pg/spore), *Penicillium expansum* (7.4 pg/spore), *P.chrysogenum* (5 pg/spore). In the case of airborne moulds of the genus *Aspergillus* or *Alternaria*, the ergosterol may prove difficult to detect. However, studies have shown large variety of species among the moulds found in air, which provides an argument for the possibility of estimating mould numbers based on ergosterol measurements. Another such argument is the presence of mould in the air in the form of both spores and mycelium fragments, as the ergosterol concentration has been shown to be 1,000 times higher in mycelium than in spores. Previous research has shown that mycelium fragments make a significant quantitative contribution to the pool of all forms present in the air and also have more powerful allergic effects than airborne spores (Górny et al. 2002). Considering the aforementioned facts, it can be concluded that the ergosterol content in the air may be measurable even at a low level of contamination of the order of 10^2^ cfu/m^3^.

No significant differences (*p* > 0.05) were found between the concentration of ergosterol in living and non-living mycelium, except of mould species *Cladosporium cladosporioides* and *Penicillium expansum*. Similar conclusions were reached by Mille-Lindblom et al. (2004) in a study of the biomass of fungi present in the soil. They found that after the death of a fungus the ergosterol content fell by a maximum of 34 % after 2 months’ storage. In the present work, the differences in the ergosterol content in living and dead mycelium amounted to a maximum of 20–33 % after 2 weeks’ storage.

Mille-Lindblom et al. (2004) also noted the significant effect of photochemical degradation on the level of ergosterol, which may decrease by as much as 43 % under the action of light after only 24 h. Light may be of key significance in the reduction of ergosterol in the cells of microorganisms present in outdoor air. A known phenomenon is photochemical reduction of ergosterol leading to degradation of the double bond in the ergosterol molecule and conversion of the compound to ergocalciferol (Holick 1984; Newell et al. 1988). Particularly, strong effects are produced by UV radiation (Schwadorf and Müller 1989), which also may be active in the atmospheric environment. It must, therefore, be borne in mind that determining the level of mould contamination of outdoor air based on ergosterol concentration may give lower results than those obtained by analysis using the culture method. These hypotheses are confirmed by the results obtained in the present study relating to comparative analysis of mould contamination of the air in different environments as measured using the culture method and by ergosterol determination. In outdoor air samples in which high mould numbers were found by the culture method (5.4 × 10^3^ cfu/m^3^), there was observed to be a lower concentration of ergosterol (1.0 ng/m^3^) compared with highly mould-infested rooms (3.1–3.6 × 10^3^ cfu/m^3^), which had an ergosterol content of 1.4–2.6 ng/m^3^.

The ergosterol content in the spores is low, as was shown in our work, probably due to the limited number of mitochondria in spores (ergosterol is mainly present in mitochondrial membranes; Seitz et al. (1979). The features of spore coverings, such as their significant thickness, the presence of pigments, and the chemical composition, protect the spores from the action of light, and hence, these forms are resistant to UV radiation. To obtain a significant effect in spores reducing, it is necessary to use a 100 times higher dose of UV radiation than in the case of mycelium. In atmospheric air, UV radiation primarily degrades the ergosterol present in mycelium fragments, having a negligible effect in reducing ergosterol content in spores. This explains the difference in the results obtained for the atmospheric air (lower level of ergosterol relative to the culture method) in comparison with the indoor air in mould-infested rooms, where there is lower UV effect, and the moulds occur in the form of numerous fragments of living mycelium (correlation between ergosterol content and cfu/m^3^). In rooms in which there was active development of moulds on building partitions or other objects, a significant airborne contribution may come from fragments of living mycelium, in which case the ergosterol concentration may be high and overestimated compared with the results obtained by the culture method. This situation was found in residential buildings where mould was observed on the walls, and in museum storerooms containing mould-infested historical objects. The highest level of ergosterol concentration in the air was found in residential premises; in 10 buildings it ranged from 1.4 to 6.4 ng/m^3^ air.

Environmental analysis also showed that in the case of high airborne mould levels, exceeding 10^3^ cfu/m^3^, the ergosterol concentration recorded was always above 1 ng/m^3^.

Based on comparison of the results of analysis of ergosterol and of moulds numbers determined by the culture method, it can be assumed that a concentration of ergosterol above 1 ng/m^3^ corresponds to a number of moulds of approximately 10^3^ cfu/m^3^. It should nonetheless be borne in mind that ergosterol content correlates above all with the types of mould and morphological forms present in the air, not only with the number of moulds. Tests should be carried out in the future to include analysis of ergosterol in a larger number of premises with more varied levels of moulds infestation (the present study concerned 42 rooms with moulds contamination at a level of 10^2^–10^3^ cfu/m^3^). Future studies should also include a detailed analysis of taxonomic units and morphological forms in the investigated environments.

## Conclusions

The presented study confirms the possibility of using ergosterol as the estimated indicator of mould’s presence in the air in the form of spores and fragments of live and inactive mycelium. However, it should be considered, that, depending on the morphological form of moulds presented in the air and the kind of environment (indoor and outdoor air) indicator may show the different result than obtained in culture method. Therefore, the determination of the airborne moulds amount by multiple methods should be performed, which would provide various information such as the number of living cells and the total microbial biomass in the air. An important issue is the development and validation of the air sampling methods for microbial contamination analysis, which are the first and key steps of the environment assay.
